# The CXCL12/CXCR4 Axis Plays a Critical Role in Coronary Artery Development

**DOI:** 10.1016/j.devcel.2015.03.026

**Published:** 2015-05-26

**Authors:** Sarah Ivins, Joel Chappell, Bertrand Vernay, Jenifer Suntharalingham, Alexandrine Martineau, Timothy J. Mohun, Peter J. Scambler

**Affiliations:** 1 Developmental Biology of Birth Defects, UCL Institute of Child Health, 30 Guilford Street, London WC1N 1EH, UK; 2 Developmental Biology Division, MRC National Institute for Medical Research, The Ridgeway, Mill Hill, London NW7 1AA, UK

## Abstract

The chemokine CXCL12 and its receptor CXCR4 have many functions during embryonic and post-natal life. We used murine models to investigate the role of CXCL12/CXCR4 signaling in cardiac development and found that embryonic *Cxcl12*-null hearts lacked intra-ventricular coronary arteries (CAs) and exhibited absent or misplaced CA stems. We traced the origin of this phenotype to defects in the early stages of CA stem formation. CA stems derive from the peritruncal plexus, an encircling capillary network that invades the wall of the developing aorta. We showed that CXCL12 is present at high levels in the outflow tract, while peritruncal endothelial cells (ECs) express CXCR4. In the absence of CXCL12, ECs were abnormally localized and impaired in their ability to anastomose with the aortic lumen. We propose that CXCL12 is required for connection of peritruncal plexus ECs to the aortic endothelium and thus plays a vital role in CA formation.

## Highlights

• *Cxcl12* and *Cxcr4* mutants lack intra-ventricular coronary arteries
• Coronary artery stem formation is impaired in *Cxcl12* mutants
• Peritruncal blood vessels fail to penetrate the aortic endothelium
• CXCL12 signaling is required for anastomosis of peritruncal endothelial cells

## Graphical Abstract

**Figure:**
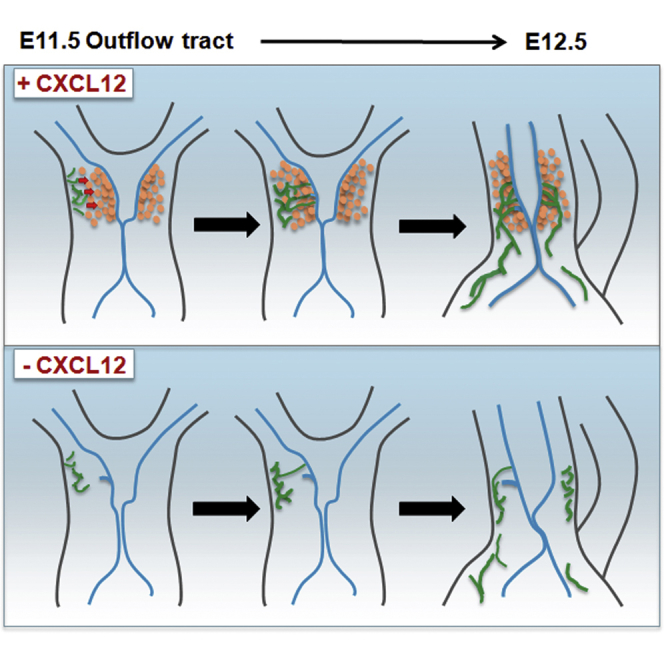

## Teaser

Coronary artery stem formation depends on the establishment of connections between the peritruncal capillary network and the lumen of the developing aorta. Ivins et al. have shown that in mice deficient for the chemokine CXCL12, the formation of such connections is disrupted, leading to severe coronary artery defects.

## Introduction

Formation of the coronary vascular system coincides with a period of rapid expansion in cardiac size between embryonic day (E) 11.5 and E13.5. Myocardial growth requires enhanced oxygen delivery and triggers an influx of endothelial cells (ECs) that undergo vasculogenesis to form a capillary plexus (Mikawa and Fischman, 1992). Coronary ECs invade the myocardium from a variety of sources, including the pro-epicardium (Katz et al., 2012), endocardium (Wu et al., 2012), and sinus venosus (Red-Horse et al., 2010; Tian et al., 2013b). Coronary lineage tracing has been utilized in a recent study to shown both complementary (sinus venosus and endocardium) and cardiac-wide (pro-epicardium) distribution of coronary ECs from these three sources (Chen et al., 2014b). Extensive angiogenic remodeling of the vascular plexus takes place from E14.5, giving rise to the mature coronary vascular system (Kattan et al., 2004; Morabito et al., 2002).

Normally, two major coronary arteries (CAs) arise from the aorta, with their stems positioned at the left and right aortic valve sinuses. The origins of the CA stems were first elucidated in avian embryos, where it was found that they form from an ingrowth of ECs into the aorta (Bogers et al., 1989; Waldo et al., 1990). Further investigation showed that endothelial strands grow into the aorta from a ring of capillaries encircling the outflow tract (peritruncal plexus) and penetrate the aortic sinuses at multiple sites. By mechanisms that are unknown so far, the peritruncal vessels persist and remodel at only two of these sites, ultimately forming the CA stems (Aikawa and Kawano, 1982; Ando et al., 2004; Poelmann et al., 1993; Ratajska and Fiejka, 1999; Waldo et al., 1990). This ingrowth model of CA stem formation has recently been confirmed in mice (Tian et al., 2013a). The molecular signals underlying CA stem and ostia formation have yet to be fully elucidated, although factors such as vascular epidermal growth factor (VEGF), fibroblast growth factor 2 (FGF-2), and platelet-derived growth factor β (PDGFβ) have been found to be required (Tomanek et al., 2006, 2008; Van den Akker et al., 2008), indicating that multiple growth factors and signaling molecules are involved in this tightly orchestrated process. In particular, a role for chemokine activity in directing EC migration to the aortic sinuses has been suggested (Waldo et al., 1990).

CXCL12 and the G-protein-coupled cell surface receptor CXCR4 are a chemokine ligand and receptor pair that have been shown to regulate cell behavior and migration in numerous contexts (Kiefer and Siekmann, 2011; Teicher and Fricker, 2010). During development, CXCL12 drives migration of cells expressing CXCR4, including ECs (Li et al., 2013) and neural crest (Belmadani et al., 2005; Escot et al., 2013; Theveneau et al., 2010), while knockout studies have shown that mice lacking either CXCL12 or CXCR4 die in utero or perinatally, with identical defects in hematopoiesis and cerebellar development as well as a cardiac ventricular septal defect (VSD) (Nagasawa et al., 1996, Ma et al., 1998, Tachibana et al., 1998, Zou et al., 1998). CXCL12-CXCR4 signaling has been shown to play a significant role in angiogenesis in the gut (Ara et al., 2005; Tachibana et al., 1998), kidney (Takabatake et al., 2009), and brain, indicative of an organ-specific role in vascularization (Kiefer and Siekmann, 2011).

We have now identified a critical role for CXCL12-CXCR4 signaling in the development of CAs in mice. By examining the hearts of *Cxcl12* and *Cxcr4* mutant embryos late in development, we have uncovered a severe phenotype, with complete absence of intra-ventricular CAs and abnormalities in the number and position of coronary ostia. Detailed expression analysis and confocal microscopy in *Cxcl12* mutants have enabled us to trace the origins of the defects to the development of the peritruncal plexus from which the CA stems originate. Our data suggest a model whereby CXCL12 secreted by cells of the aortic wall acts as a chemoattractant for CXCR4-positive peritruncal ECs and is required for anastomosis of peritruncal ECs with the lumen of the aorta. Impairment of this process in *Cxcl12* or *Cxcr4* mutants results in failure of arterialization of the main coronary plexus.

## Results

### Severe Defects in CA Development in *Cxcl12*- and *Cxcr4*-Mutant Hearts

**Figure 1:**
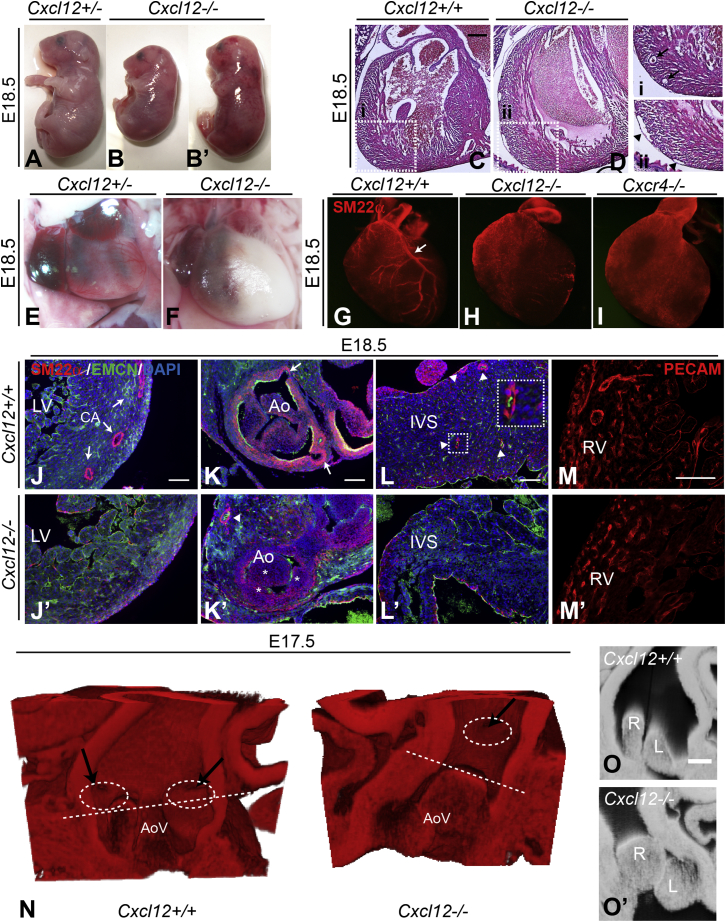
Mutation of *Cxcl12* Causes Severe CA Defects (A–B’) Control (A) and *Cxcl12*^*−/−*^ (B and B’) E18.5 embryos; note the small size of the null embryo in (B) and the hemorrhagic appearance of the embryo in (B’). (C and D) H&E-stained E18.5 heart sections; boxed areas in control (C) and *Cxcl12* null (D) are shown enlarged to the right (boxes i and ii). Arrows in (i) indicate intra-ventricular CAs; arrowheads in (ii) indicate dilated veins in the null. (E and F) Frontal view of E18.5 *Cxcl12*^*+/−*^ (E) and *Cxcl12*^*−/−*^ (F) hearts. (G–I) Whole-mount SM22α antibody staining of CAs at E18.5. Arrow indicates left CA in a wild-type control (G); *Cxcl12*^*−/−*^ (H) and *Cxcr4*^*−/−*^ (I) hearts lack CAs. (J–M’) Analysis of CAs in E18.5 heart sections: SM22α marks CAs, EMCN labels endocardium, and nuclei are counterstained with DAPI. CAs are indicated by arrows in wild-type myocardium (J) but are absent in *Cxcl12* null myocardium (J’). Control coronary ostia are positioned in the aortic valve sinuses (arrows in K). Arrowhead in (K’) shows a single CA proximal to the aorta (Ao) in the null, asterisks indicate thickened aortic valve leaflets. Arrowheads in (L) indicate arteries in control IVS; inset shows enlargement of one artery; null IVS (L’) lacks vessels and appears necrotic. (M and M’) PECAM antibody staining shows vascular plexus in control and *Cxcl12*^*−/−*^ hearts. EMCN, endomucin; LV, left ventricle; RV, right ventricle. (N–O’) HREM on E17.5 hearts. (N) shows 3D reconstructions (Imaris) of control and *Cxcl12* null aorta walls (cut away); arrows and dashed circles indicate coronary ostia, and dashed lines indicate the top of aortic valve (AoV) leaflets. A single ostium was observed in the null. Two-dimensional (2D) slices through the aorta (O and O’) show thickened left and right AoV leaflets (L and R, respectively) in the null (O’). Scale bars represent 250 μm in (C) and (D) and 100 μm in (J)–(M). See also Figure S1 and Movie S1.

We carried out interbreeding of *Cxcl12* heterozygous animals in order to investigate the cardiovascular phenotype of *Cxcl12* null embryos. Despite the reported 50% lethality of *Cxcl12* mutation at E18.5 (Nagasawa et al., 1996), we were able to recover viable mutant embryos at this stage at approximately Mendelian ratios. *Cxcl12* mutants were stunted in growth to a variable degree, and a number of them showed evidence of hemorrhaging (compare Figure 1A with Figures 1B and 1B’). Furthermore, the hearts of all mutant embryos appeared pale and enlarged compared to those of controls (Figures 1E and 1F). H&E staining of mutant heart sections showed a lack of large vessels in the *Cxcl12*^−/−^ ventricular myocardial wall and the presence of dilated veins (compare Figures 1C and 1D).

In order to visualize coronary vessels more clearly, whole E18.5 control and *Cxcl12* mutant hearts were stained for smooth muscle expression using anti-SM22α antibody (Figures 1G and 1H). Strikingly, we observed that left and right CAs were not visible in any *Cxcl12* mutants (Figure 1H; Figures S1A–S1C). An identical phenotype was observed in *Cxcr4* mutant hearts (Figure 1I). Coronary veins were present in *Cxcl12* mutants (Figures S1A–S1C) and in *Cxcr4* mutants (data not shown). Thus, the most severe coronary vessel defects in the *Cxcl12*/*Cxcr4* mutant hearts appeared to be artery specific. Immunolabeling of sections confirmed the absence of CAs in E18.5 *Cxcl12* mutant hearts in the ventricular walls (compare Figures 1J and 1J’) and inter-ventricular septum (IVS) (Figures 1L and 1L’). Although single CAs proximal to the aorta were observed in *Cxcl12* mutants, ostia were not located in the normal position in the aortic valve sinuses (compare Figures 1K and 1K’). We noted that these abnormal proximal CAs were blood filled and patent to ink in a further set of mutants (data not shown). Staining for the widely expressed endothelial marker PECAM revealed the presence of a comparatively normal capillary plexus in null hearts, albeit lacking larger vessels (compare Figures 1M and 1M’), showing that the lack of well-developed CAs in *Cxcl12* null hearts cannot be attributed to a lack of ECs in the myocardium.

The aortic valve leaflets also appeared profoundly abnormal in *Cxcl12* mutants (Figure 1K’). In order to visualize the artery and valve defects more clearly, high-resolution episcopic microscopy (HREM) (Mohun and Weninger, 2012) was carried out on *Cxcl12* null (n = 3) and wild-type (n = 3) hearts (Figures 1N–1O’; Movie S1). In the null hearts, the aortic valve leaflets appeared thickened and were positioned at differing levels within the aorta (Figure 1O’). Similar defects were observed in the pulmonary valves (Movie S1). Each HREM-examined null heart had a single, distally positioned ostium, with a short section of proximal CA that failed to branch into the ventricles and terminated in the IVS (Figures 1N and S1D–S1H’; Movie S1). In one mutant heart, the ostium was also displaced ventrally (Figure 1N). Thus, all *Cxcl12* null hearts examined combined a lack of distal CAs with severe semi-lunar valve (SLV) defects and a failure of proximal artery/CA stem formation on at least one side.

### Expression of *Cxcl12* and *Cxcr4* during Cardiac Vascularization

Given the CA phenotype described earlier, we wanted to examine cardiac expression of both *Cxcl12* and *Cxcr4* in detail from E11.5 onward. This developmental stage was selected as hearts are avascular before E10.5, while E11.5 marks the beginning of the expansion of the coronary plexus that reportedly invades the ventricular myocardium from E12.5 to E14.5 (Red-Horse et al., 2010; Tian et al., 2013b).

**Figure 2:**
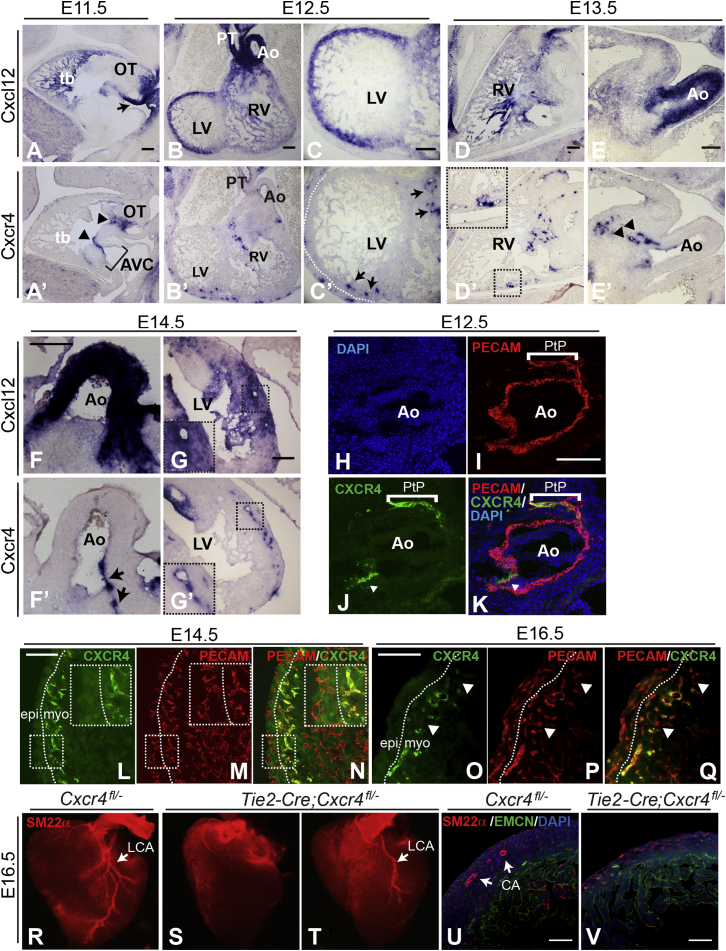
Endothelial Expression of *Cxcr4* Is Required for Coronary Arteriogenesis (A–G’) *Cxcl12* and *Cxcr4* expression in serial sections of E11.5–E14.5 wild-type hearts (in situ hybridization). (A and A’) Sagittal sections at E11.5. Arrow in (A) indicates *Cxcl12* in the inferior wall of the OT; arrowheads in (A’) show *Cxcr4* in the lining of OT and AVCs (bracketed). (B–C’) Transverse sections at E12.5. (B and C) *Cxcl12* is highly expressed in OT vessels and left ventricle (LV) and at a lower level in the right ventricle (RV). Arrows in (C’) indicate *Cxcr4*-positive vessels in the ventricular wall; dotted line in (C’) delineates ventricle wall. (D–E’) Sagittal sections at E13.5. Inset in (D’) shows enlargement of *Cxcr4*-positive vessels in the ventricle wall (boxed); arrowheads in (E’) indicate *Cxcr4* expression in the peritruncal plexus (PtP); note strong aortic *Cxcl12* expression in the consecutive section in (E). (F–G’) Transverse sections at E14.5. Arrows in (F’) indicate *Cxcr4* in the maturing LCA and ostium. Boxed area shown enlarged in (G and G’) shows *Cxcr4* and *Cxcl12* expression in a large coronary vessel. Ao, aorta; PT, pulmonary trunk; tb, trabeculated myocardium. (H–K) Transverse sections through wild-type E12.5 aorta. CXCR4/PECAM co-localization is shown in endothelial cells of the peritruncal plexus (bracketed). Note the lack of CXCR4 expression in the endothelial lining of the aorta. Arrowheads in (J) and (K) indicate aortic valve endothelium. (L–Q) CXCR4/PECAM co-localization in intra-myocardial ECs. Insets show enlargements of boxed regions; dotted lines delineate sub-epicardial (epi) and myocardial (myo) compartments of ventricular wall. CXCR4 is expressed in myocardial, but not sub-epicardial, ECs at E14.5 (L–N, maximum intensity z-projections of confocal stacks). CXCR4 is reduced or absent in smaller myocardial vessels at E16.5 (O–Q, arrowheads). (R–V) Whole-mount SM22α staining of E16.5 *Tie-2Cre*;*Cxcr4*^*fl/−*^ and control hearts (ventral views). Null in (S) lacks CAs; arrow in (R) indicates LCA in a control heart. A more mildly affected conditional null (T) exhibited a truncated LCA (arrow). SM22α/EMCN/DAPI-labeled sections (U and V) show lack of CAs in *Tie2-Cre*;*Cxcr4*^*fl/−*^ ventricular myocardium. Scale bars represent 100 μm. See also Figure S2.

Expression of *Cxcl12* and *Cxcr4* was analyzed by carrying out in situ hybridization on serial sections. At E11.5, *Cxcl12* was highly expressed in the wall of the outflow tract (OT) and at a lower level in the ventricular walls and trabeculated myocardium, while *Cxcr4* expression was localized to the endocardial lining of the OT and atrioventricular cushions (AVCs) as previously reported (Sierro et al., 2007), (Figures 2A and 2A’). At E12.5, a number of vessels expressing *Cxcr4* were observed, restricted to the myocardial layer of the ventricle walls and the peritruncal region (Figures 2B’ and 2C’; data not shown). The sites of *Cxcr4* expression correlated with the emerging intra-myocardial coronary plexus (Tian et al., 2013b). *Cxcr4* was also expressed in the endothelium and underlying mesenchyme of the aortic and pulmonary valves, as well as in the AV valves from E12.5 onward (data not shown). High levels of *Cxcl12* expression were seen in the walls of the aorta and pulmonary trunk at this developmental stage (Figure 2B), as well as in the left ventricle, with lower levels in right ventricular myocardium (Figures 2B and 2C).

Expression of both genes was maintained at E13.5 (Figures 2D–2E’), with increasing numbers of *Cxcr4*-expressing vessels observed in the ventricular myocardial walls by E14.5 (Figure 2G’). At E13.5, *Cxcr4* expression was also detected in peritruncal ECs, coinciding with a region of strong *Cxcl12* expression in the aortic wall (Figures 2E and 2E’), while from E14.5 onward, *Cxcr4* expression was observed in the maturing CAs and ostia (Figure 2F’; data not shown). *Cxcl12* expression remained at high levels in both aorta and ventricular myocardium at E14.5 (Figures 2F and 2G), decreasing at later stages (data not shown). In addition, from E14.5, larger intra-myocardial vessels were observed to express both *Cxcr4* and *Cxcl12* (Figures 2G and 2G’; data not shown). *Cxcr4* was not expressed in sub-epicardial ECs or vessels at this or any of the stages examined.

**Figure 3:**
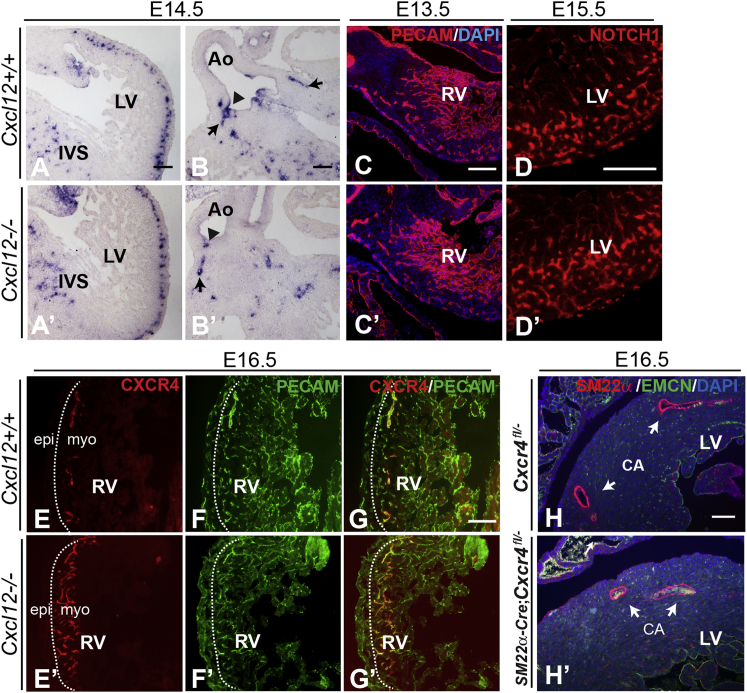
ECs in *Cxcl12*-Mutant Hearts Are Normally Localized, but Fail to Downregulate CXCR4 at E16.5, while Loss of CXCR4 in the Smooth Muscle Lineage Does Not Affect CA Development (A–B’) In situ hybridization analysis shows that *Cxcr4* expression is unaltered in E14.5 *Cxcl12*^*−/−*^ myocardial vessels (A’) compared to control (A). Arrowheads in (B) and (B’) indicate coronary ostia; arrows indicate proximal CAs. LV, left ventricle; Ao, aorta. (C and C’) PECAM antibody staining shows that ECs are localized normally in *Cxcl12*^*−/−*^ ventricular myocardium at E13.5. RV, right ventricle. (D and D’) NOTCH1 expression in control and *Cxcl12*^*−/−*^ hearts. (E–G’) CXCR4/PECAM antibody labeling of E16.5 wild-type and *Cxcl12*^*−/−*^ heart sections; CXCR4 is co-expressed with PECAM in all intra-myocardial (myo) vessels in the null (E’–G’) in contrast to its more restricted expression in the control (E–G). Dotted lines delineate sub-epicardial (epi) and intra-myocardial regions of ventricle wall. (H and H’) Conditional deletion of *Cxcr4* in the smooth muscle lineage using *SM22α-Cre* does not affect CA development. SM22α/EMCN/DAPI labeling shows normal CAs (arrows) present in E18.5 *SM22a*;*Cxcr4*^*fl/−*^ heart sections in (H’). Scale bars represent 100 μm. See also Figure S3.

Antibody labeling of OT sections confirmed the expression of CXCR4 in peritruncal plexus ECs (Figures 2H–2K). While anti-PECAM antibodies labeled ECs of the peritruncal plexus and aortic lumen (Figure 2I), CXCR4 staining was restricted to peritruncal ECs and aortic valve endothelium (Figure 2J). Co-staining of E14.5 heart sections with CXCR4 and PECAM antibodies corroborated the aforementioned in situ data, further showing that ventricular expression of CXCR4 was confined to ECs localized in the myocardium (Figures 2L–2N). By E16.5, CXCR4 expression was reduced or extinguished in most of the intra-myocardial capillary plexus, being retained mainly in larger vessels (Figures 2O–2Q; Figure 3E). Thus, CXCR4 expression appeared to be linked to the re-modeling/maturation of the coronary network, declining as larger vessels developed.

To test whether deletion of *Cxcr4* in the endothelial lineage alone was sufficient to recapitulate the CA phenotype found in *Cxcl12*/*Cxcr4* mutants, we used the *Tie2-Cre* transgene and a floxed *Cxcr4* allele (Nie et al., 2004) (Figures 2R–2V). Of seven E16.5 *Tie2-Cre;Cxcr4*^*fl/−*^ embryos examined by whole-mount SM22α immunolabeling, four were observed to display the same severe CA phenotype as *Cxcl12* and *Cxcr4* nulls (Figure 2S). Two had a milder phenotype, with CAs that followed a normal course over the left and right ventricles but that appeared shorter and less branched than in control hearts (particularly the left CA [LCA]; one example shown in Figure 2T), and one appeared normal (data not shown). Sections from another severely affected conditional null showed that, as well as lacking intra-ventricular CAs (Figure 2V), SLV defects were also present, along with a single, distally positioned ostium (Figures S2A’ and S2B’). Thus, EC-specific deletion of *Cxcr4* recapitulated the CA phenotype observed in *Cxcl12* nulls.

As a proportion of the conditional mutants appeared to be less severely affected than full nulls, we investigated the efficiency of the Cre recombinase in *Tie2-Cre;Cxcr4*^*fl/−*^ hearts at E15.5. In one of four conditional null hearts examined by in situ hybridization, strong *Cxcr4* expression was still observed (Figure S2D), indicating a failure of Cre-mediated recombination in the endothelium. Therefore, inefficient recombination of the *Cxcr4* floxed allele by the *Tie2*-driven Cre recombinase likely explains the reduced penetrance of the CA phenotype in the conditional nulls.

### CA Maturation Failure in *Cxcl12* Mutants Is Not Caused by Defective EC Localization, Arterial Fate Specification, or vSMC Recruitment

As described earlier, CXCR4 is expressed in ECs located within the compact myocardium of the ventricles. ECs localized in the ventricular myocardium are fated to form capillaries and arteries rather than veins (Red-Horse et al., 2010; Tian et al., 2013b; Wu et al., 2012). To investigate the possible mechanism(s) underlying the failure of CA formation in the *Cxcl12* nulls, we first examined the localization of *Cxcr4*-expressing ECs in *Cxcl12* mutant hearts. However, this was found to be normal, as determined by in situ hybridization at E14.5 (compare Figures 3A and 3A’). The main difference observed at this stage was the lack of any larger bore vessels on the left side of the aorta (a single ostium was observed on the right side) (compare Figures 3B and 3B’), consistent with the defects we observed at later stages. Similarly, immunolabeling with PECAM antibody at E13.5 (Figures 3C and 3C’) and E14.5 (data not shown) did not reveal abnormalities in the distribution of coronary ECs throughout the myocardial and sub-epicardial layers of *Cxcl12* mutant hearts. Thus, CXCL12-CXCR4 signaling is not required for myocardial invasion by coronary ECs.

To test the possibility that the myocardially localized ECs were not correctly specified for arterial differentiation, we used NOTCH1 as a marker of arterial fate (del Monte et al., 2011). However, we found that NOTCH1 was expressed normally in *Cxcl12* mutant hearts at E15.5 (Figures 3D and 3D’) and E16.5 (data not shown), consistent with correct arterial specification of coronary ECs.

These data indicated that coronary ECs in *Cxcl12* mutants are both correctly localized and specified for arterial differentiation; therefore, the lack of CA formation is likely caused by the failure of the coronary plexus to mature/re-model. Our observation that CXCR4 expression becomes restricted mainly to larger coronary vessels between E14.5 and E16.5 (Figures 2O–2Q) suggested that CXCR4 levels may serve as an indicator of the state of maturation of the coronary plexus. Therefore, we examined CXCR4 expression in *Cxcl12* mutant hearts at E16.5 and found that, in contrast to control hearts (Figures 3E–3G), small CXCR4/PECAM co-stained vessels were visible throughout the myocardium of mutant ventricles (Figures 3E’–3G’) and IVS (data not shown). This was similar to the pattern observed in E14.5 wild-type hearts (Figure 2N), supporting the hypothesis that the vascular plexus is retained in an immature state in mutant hearts. Quantification of the number of ECs in mutant hearts confirmed the increase in the proportion of CXCR4-expressing ECs but showed that there was no difference in the overall number of ECs compared to controls (data not shown).

Recruitment of mural cells (pericytes and vascular smooth muscle cells [vSMCs]) to newly formed vessels is required to stabilize, remodel, and mature the primary vasculature. As expression of CXCR4 has been reported in both pericytes and vSMC progenitors (Song et al., 2009; Zernecke et al., 2005; Petit et al., 2007), we wanted to examine the potential role of the CXCR4/CXCL12 axis in the recruitment of mural cells to the coronary vasculature. Therefore, *SM22α*-driven Cre recombinase was used to inactivate *Cxcr4* in vSMCs and pericytes (Anastasia et al., 2014). However, CAs were found to have developed normally in all conditional nulls examined at E18.5 (Figure 3H’; n = 8), although a high percentage of SLV defects was observed (87.5% of conditional nulls) (Figures S3A’ and S3B’). Coverage of CAs with vSMCs (as analyzed using SM22α/PECAM expression) was unaffected in *SM22α-Cre;Cxcr4*^*fl/−*^ hearts (Figure S3C’). Therefore, a lack of mural cell recruitment does not appear to be the underlying cause of the failure of coronary plexus maturation in *Cxcl12* null hearts.

### Confocal Analysis of Peritruncal Plexus Development

Another requirement for maturation of the intra-ventricular coronary plexus is blood flow into the coronary ostia; development of the tunica media of the arteries has been shown to proceed in a proximo-distal direction at the onset of coronary flow (Ando et al., 2004; Ratajska and Fiejka, 1999; Vrancken Peeters et al., 1997). Therefore, defective development of the CA stems/ostia, preventing blood flow into the coronary vessels, would abrogate the maturation process. Absent coronary ostia in *Cxcl12* null hearts coupled with expression of *Cxcr4* and *Cxcl12* in peritruncal ECs and aortic wall, respectively (discussed earlier) suggest a potential role for CXCL12-CXCR4 signaling in CA stem formation.

**Figure 4:**
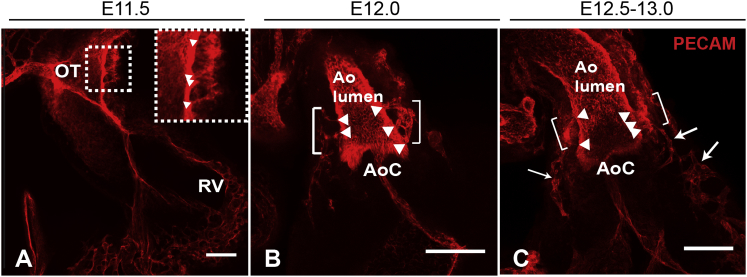
Development of the Peritruncal Plexus (A−C) Confocal microscopic images of whole-mount PECAM-labeled hearts (maximum intensity z projections of confocal stacks) showing development of the peritruncal plexus from E11.5 to E13.0. Inset in (A) is an enlargement of the boxed region, showing processes from an EC cluster contacting the OT lumen at E11.5. At E12.0 (B) and E12.5–E13.0 (C), the peritruncal plexus is seen on both sides of the aorta (Ao), just distal to the aortic cushions (AoC). Arrowheads indicate points of contact between ECs and the aortic lumen. Vessels connecting the peritruncal plexus with the main coronary plexus are not observed until approximately E12.5–E13.0 (arrows in C). RV, right ventricle. Scale bars represent 100 μm. See also Figure S4.

**Figure 5:**
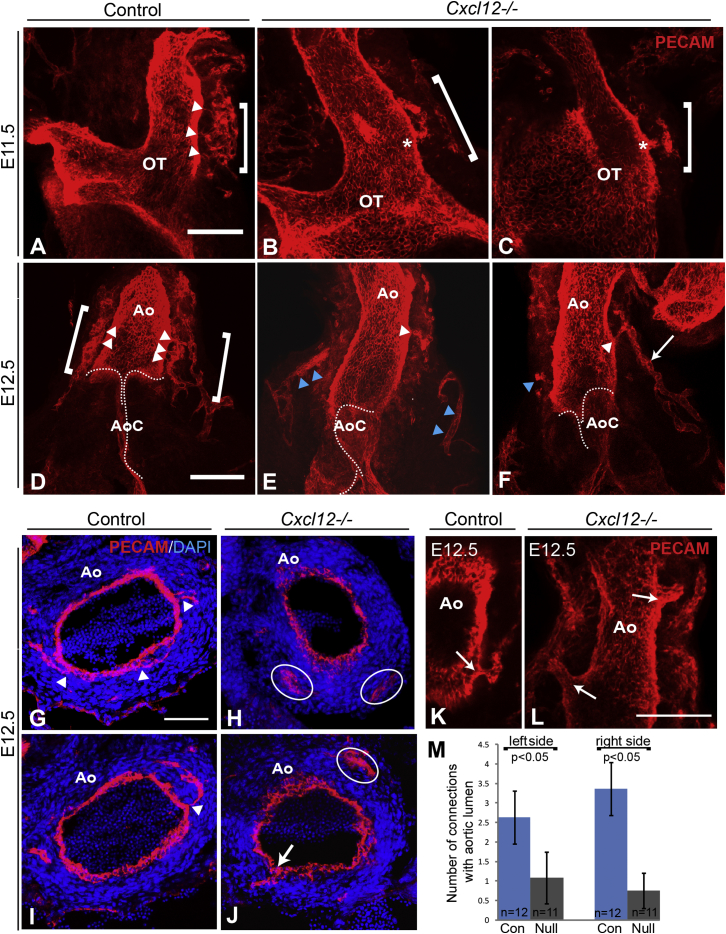
Defective Anastomosis of the Peritruncal Plexus in *Cxcl12*-Null Hearts (A–F) Confocal microscopic images of E11.5–E12.5 whole-mount PECAM-labeled control and *Cxcl12*^*−/−*^ hearts showing the OT/aorta (Ao) and peritruncal plexus (bracketed region). (A–C) Images at E11.5. Arrowheads indicate multiple points of contact between ECs and OT in the control, shown in (A). In contrast, EC clusters localized in the sub-epicardial region of *Cxcl12* nulls lack connections with the OT lumen, shown in (B and C); asterisks indicate vessels that appear to sprout from the OT lumen of nulls. (D–F) At E12.5, *Cxcl12* nulls (E and F) show reduced/absent peritruncal EC anastomoses (arrowheads) on both sides of the aorta, compared to controls (D); disconnected EC strands/clusters (blue arrowheads in E and F) and sub-epicardially localized vessels (arrow in F) are also observed in nulls. Dotted lines delineate the edges of the aortic cushions (AoC). (G–J) PECAM/DAPI-stained sections (20 μm) show anastomoses (arrowheads, G and I) formed by peritruncal ECs in control hearts. Null sections lack anastomoses, and ECs are clustered away from the aortic lumen (white ovals in H and J). Arrow in (J) indicates a possible sprout from the aortic lumen. (K and L) Confocal microscopic images of whole-mount PECAM-labeled E12.5 hearts show vessels sprouting from the aortic lumen (arrows) in control (K) and null (L) hearts. Both proximal and distal lumen sprouts can be observed in the null. (M) Comparison of numbers of peritruncal EC anastomoses formed in E12.5 controls (Con; blue columns) versus *Cxcl12* nulls (gray columns). Proximal (but not distal) sprouts from the aortic lumen were included in the data. Data are presented as mean ± SD. All of the aforementioned images in (A)–(L) are maximum intensity z projections of confocal stacks. Scale bars represent 100 μm. See also Figure S4.

In order to better understand a potential role for CXCL12-CXCR4 signaling in this process, we first made a detailed study of peritruncal plexus formation from E11.5 onward, using confocal microscopy of optically cleared whole-mount PECAM-stained wild-type hearts. Previous studies have not investigated peritruncal plexus formation at murine developmental stages prior to E12.5. At E11.5, we observed isolated clusters of ECs in the dorsal part of the OT wall anterior to the cushions, with multiple processes contacting the OT lumen (boxed region in Figure 4A; Figure 5A). The only other coronary ECs observed at this stage were in the sub-epicardial region of the ventricles (data not shown), consistent with previously reported observations (Tian et al., 2013b). Therefore, peritruncal plexus formation *precedes* development of the main intra-myocardial coronary plexus. By E12.5, the peritruncal plexus had expanded around the proximal part of the aorta, anterior to the cushions; clusters of interconnected EC strands were now observed on either side of the aorta, located close to the lumen as well as in the sub-epicardial compartment, again with multiple processes forming anastomoses with the aortic lumen (Figures 4B and 5D). At this stage, the peritruncal plexus was still not continuous with the developing intra-ventricular plexus, but inter-connecting vessels were observed at the base of the aorta at a slightly later stage (approximately E12.5–E13.0; arrows in Figure 4C). By E13.5, the peritruncal plexus had taken on a more organized, mature appearance, with some of the EC strands appearing wider and lumenized (Figure S4A). Multiple points of contact were still retained between the EC strands and both sides of the aortic lumen at various points level with, and distal to, the aortic valve, as previously described (Ando et al., 2004; Poelmann et al., 1993; Ratajska and Fiejka, 1999; Tian et al., 2013a; Waldo et al., 1990), and the peritruncal plexus was now fully connected to the main coronary capillary network. A day later, at E14.5, multiple aorta-contacting vessels were still observed in some hearts (e.g., on the right side of the aorta, shown in Figure S4D, in contrast with the major vessel on the left side), but by E15.5, these vessels had resolved into a single major lumenized vessel on either side of the aorta, generally positioned at the level of the aortic valve (data not shown).

### Peritruncal ECs in *Cxcl12*-Null Hearts Are Impaired in Their Ability to Anastomose with the Aortic Lumen

Peritruncal plexus development was then examined in *Cxcl12* mutants. At E11.5, striking peritruncal plexus abnormalities were observed in five of seven mutants examined (71%). In the examples shown in Figures 5B and 5C, clusters of ECs were observed in the sub-epicardial layer of the OT (bracketed regions); however, the ECs lacked processes contacting the aortic lumen, in contrast to controls (n = 6; Figure 5A). In addition, in each mutant, a short vessel protruded from the lumen of the OT (asterisks in Figures 5B and 5C). Dramatic differences were also observed at E12.5. Where EC strands in control aortas were observed to form multiple anastomoses on the left and right sides of the lumen (n = 12; Figure 5D), all *Cxcl12* mutants (n = 11) were characterized by reduced or absent peritruncal EC connections. In many cases, a single contacting vessel/EC strand was present (7 of 11), and peritruncal EC strands/clusters were frequently localized solely in the sub-epicardial layer of the aorta (Figures 5E and 5F). Similar results were observed at E13.5 and E14.5 (Figure S4). Sections through the OT at E12.5 confirmed that peritruncal ECs were not absent in *Cxcl12* nulls but rather were mislocalized in sub-epicardial clusters and lacked anastomotic connections with the aortic endothelium (Figures 5H and 5J). This was in contrast to controls, where anastomosing EC strands were localized in close proximity to the aortic lumen (Figures 5G and 5I). A recent study has shown that endocardial sprouts can extend from the aortic lumen and make a small contribution to the future CA stems (Chen et al., 2014a). We also observed sprouts from the aortic lumen in 33% of wild-type E12.5 hearts examined (Figure 5K); however, sprouts were wider in diameter and more frequently observed in *Cxcl12* nulls (66% of nulls; Figures 5J and 5L) and could be both proximally and much more distally positioned relative to the aortic valve (Figure 5L). Quantification of the number of peritruncal aortic connections showed a significant reduction in *Cxcl12* nulls, even when the more proximal sprouts were included (Figure 5M).

### Targeting *Cxcl12* in Ventricular Myocardium Is Insufficient to Induce CA Defects

**Figure 6:**
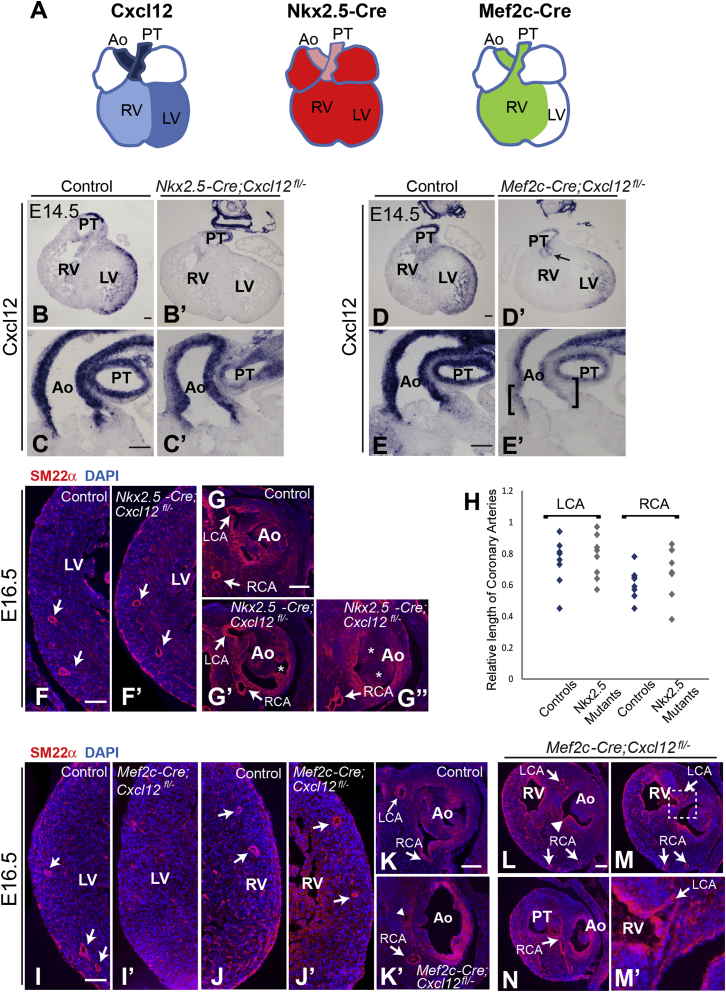
Targeting *Cxcl12* Expression in the OT Has a Severe Impact on CA Development (A) Schematic of hearts comparing regions of *Cxcl12* (blue) and Cre recombinase (as indicated by published lineage tracing) expression driven by either *Nkx2.5* (red/pink) or *Mef2c* (green). Relative expression levels are indicated by shading. Ao, aorta; PT, pulmonary trunk, LV, left ventricle; RV, right ventricle. LV, left ventricle. (B–E’) Cardiac expression of *Cxcl12* was targeted using *Nkx2.5* and *Mef2c* Cre drivers. In situ hybridizations (E14.5, heart sections) show effective targeting of *Cxcl12* expression by *Nkx2.5-Cre* in the ventricles (B’) but not the aorta (C’). Control sections are shown in (B) and (C). *Mef2c-Cre* largely abrogated *Cxcl12* expression in the right ventricle and IVS (D’); arrow in (D’) indicates residual *Cxcl12* expression at the base of the pulmonary trunk. *Cxcl12* was considerably reduced in both proximal (bracketed) and distal regions of the aorta (E’). Control sections are shown in (D) and (E). (F–G’’) Labeling with anti-SM22α antibody shows normal CAs and ostia in *Nkx2.5-Cre;Cxcl12*^*fl/−*^ hearts (arrows in F’, G’, and G’’). SLV defects were observed infrequently (G’’); asterisks indicate thickened aortic valve leaflets. Controls are shown in (F) and (G). (H) Scatter graph shows the relative length of LCAs and RCAs in control and *Nkx2.5-Cre;Cxcl12*^*fl/−*^ hearts. Relative CA length was variable in both controls and conditional nulls, with no overall difference between the two groups. (I–N) CA defects in *Mef2c-Cre;Cxcl12*^*fl/−*^ hearts; arrows indicate CAs. Absence of the LCA and left ostium (I’ and K’) was observed in several conditional nulls. Arrowhead in (K’) indicates an IVS vessel that branches from the RCA. (I) Control with CAs visible in left ventricle free wall. (J) Control with CAs in right ventricular free wall. (J’) Example of one mutant with CAs formed within right ventricle free wall. (L–N) In one *Mef2c-Cre;Cxcl12*^*fl/−*^ heart, CAs originated from the pulmonary trunk (arrow in N) and the right ventricle, just proximal to the pulmonary trunk valve (arrows in M and M’). Boxed region in (M) is shown enlarged in (M’). A third ostium was formed in the aorta of the same mutant by a CA that branched into the IVS (arrowhead in L). Scale bars represent 100 μm. See also Figure S5.

The defects we observed in *Cxcl12* nulls suggest that CXCL12 is required for anastomosis of peritruncal ECs in the aorta but not for the initial establishment of the peritruncal plexus. This role for CXCL12 signaling correlates with its high level of expression in the developing aorta. However, *Cxcl12* is also expressed in the ventricular myocardium, and we wanted to determine whether this domain of *Cxcl12* expression is also important in CA development. For example, ventricular CXCL12 could play a direct role in the remodeling of the vessel network, perhaps by modulating EC proliferation in response to coronary flow. We used two different Cre drivers in conjunction with a floxed *Cxcl12* allele (Greenbaum et al., 2013) in an attempt to differentially target aortic and ventricular myocardial expression of *Cxcl12*. *Nkx2.5*-Cre is an established driver in cardiac chamber cardiomyocytes (CMs) and is now known to be active in both first and second heart fields (FHF and SHF, respectively), although the extent and degree of expression in the SHF is likely to be reporter and genetic background dependent (Ma et al., 2008; Moses et al., 2001). For example, a recent study using the same *Nkx2.5*-*Cre* allele as that used here showed inefficient recombination in the OT (Ramsbottom et al., 2014). The *Mef2c* enhancer Cre drives recombination in anterior SHF lineages (Verzi et al., 2005) but would not be expected to effect recombination in left ventricular wall myocardium (Figure 6A). Analysis of *Cxcl12* expression in the conditional mutants showed that, as expected, *Nkx2.5*-Cre efficiently knocked down *Cxcl12* in the ventricular muscle (Figure 6B’) but had much less effect in the OT (Figure 6C’). Conversely, *Mef2c*-Cre was able to substantially reduce (but not completely abrogate) *Cxcl12* expression in the OT, particularly the proximal portion (Figure 6E’), and knock it out in the right ventricle and IVS while leaving left ventricular wall expression largely intact (Figure 6D’). Targeting in the right ventricle was not completely effective, as residual expression was observed at the base of the pulmonary trunk (arrow in Figure 6D’).

The two conditional mutants showed remarkably different phenotypes. While normal CAs and ostia were present in *Nkx2.5-Cre;Cxcl12*^*fl/−*^ mutant hearts (n = 8) (Figures 6F–6G’), with no apparent difference in CA length (Figure 6H), a range of much more severe phenotypes was observed in *Mef2c-Cre;Cxcl12*^*fl/−*^ mutant hearts (n = 9) (Table 1). CA abnormalities were seen in six mutants, including absent LCA and left ostium in four cases (Figures 6I–6K’). In addition, one mutant exhibited CAs originating from the pulmonary trunk and the portion of right ventricle immediately proximal to the pulmonary valve (Figures 6L–6N), while in another mutant, both CAs branched from the right ostium (Figures S5G–S5L). These were frequently, but not always, accompanied by SLV defects (Figures S5A–S5C), which themselves could be variable in their severity (Figures S5D–S5F). SLV defects were observed in only two *Nkx2.5-Cre;Cxcl12*^*fl/−*^ mutants (an example is shown in Figure 6G’’). The defects of CA formation and patterning observed in the *Mef2c-Cre;Cxcl12*^*fl/−*^ mutant, but not the *Nkx2.5-Cre;Cxcl12*^*fl/−*^ mutant, indicate that it is CXCL12 secreted by the aorta rather than the ventricular myocardium that plays the most significant role in CA development.

## Discussion

We have identified a crucial role for the CXCL12/CXCR4 axis in the development of the CAs. Confocal analysis of the OT at the earliest stages of CA stem development enabled us to identify abnormalities in the structure of the peritruncal plexus, the aortic capillary network from which the CA stems first develop. Peritruncal ECs were mislocalized and failed to connect to the aortic endothelium in *Cxcl12* mutants, resulting in defective CA stem development. Furthermore, although we showed *Cxcl12* and *Cxcr4* expression throughout the heart (in myocardium and ECs, respectively), conditional mutagenesis experiments identified the aorta as the source of CXCL12 critical for CA development. We propose that CXCL12 acts early in CA formation, guiding peritruncal plexus ECs toward the aortic lumen to form anastomoses, consistent with the known role of CXCL12/CXCR4 signaling in chemoattraction and guidance of collective cell movements. Failure of this process results in absent or abnormally positioned coronary stems and ostia, as well as a subsequent lack of intra-ventricular CAs in late-gestation embryos.

Our confocal analysis of the OT of developing hearts revealed the characteristic structure of the peritruncal capillary network, which connects specifically to the aorta. This appears to develop as a separate plexus from E11.5, arising from vessels in the sub-epicardial region of the aorta and initially unconnected to the main intra-ventricular coronary plexus. This contrasts somewhat with previous studies that describe a ring of coronary vessels that surround the entire outflow region and grow toward the aorta (Ando et al., 2004; Bernanke and Velkey, 2002; Waldo et al., 1990). However, this difference could be accounted for by the earlier developmental stages at which we carried out our analyses. The ability of CXCL12 to direct migration of various cell types is well documented (Belmadani et al., 2005; Escot et al., 2013; Kasemeier-Kulesa et al., 2010; Li et al., 2013; Song et al., 2009; Theveneau et al., 2010), while the reduced anastomoses and mislocalization of peritruncal ECs observed in *Cxcl12* mutants are consistent with a lack of angiogenic guidance cues in the OT resulting from deficient CXCL12 signaling. The ectopic distal positioning of those vessels that managed to form and contact the lumen in some *Cxcl12* mutants may simply indicate the failure of anastomosis in the more proximal region of the aorta and/or may point to abnormalities of proximo-distal EC migration. However, it is not yet understood how the broad expression of *Cxcl12* in the aorta could correlate with this fine-tuning of CA stem positioning; this likely involves additional molecular signals, adding another layer of complexity to the process (discussed later).

In some *Cxcl12* nulls, single, high coronary ostia were associated with short proximal CAs that failed to branch. It is possible that, in contrast to normal vessels, these result from sprouting from the aortic endothelium, as large-diameter sprouting vessels were frequently observed in *Cxcl12* mutants both proximal and distal to the aortic valve. Endothelial sprouts were also observed in normal hearts, in agreement with the recent demonstration of a limited role for aortic sprouting in normal CA stem formation (Chen et al., 2014a) and in contrast to the accepted ingrowth model. It is feasible that this mechanism is over-activated in *Cxcl12* nulls in an attempt to compensate for the lack of anastomosis from peritruncal vessels. Although this is speculative, it could be tested by lineage tracing using a Cre driver specific for the endocardial lining of the aorta, such as *Nfatc1* (Wu et al., 2012). It is also unclear why the compensatory proximal stems would be unable to connect the main coronary plexus, unless this process also requires CXCL12-dependent migration of the latter toward the aorta.

Another explanation for the failure of the proximal CA stems to grow into the ventricles in the *Cxcl12* mutants could be that disrupted blood flow resulting from the abnormally distal ostia, combined with the defective aortic valve, reduces the hemodynamic forces associated with blood entering the CAs. Shear and strain forces induced by diastolic blood flow are generally accepted to act as a necessary stimulus to CA remodeling, causing an increase in vessel lumen size and inducing smooth muscle recruitment (Jones et al., 2006; Ratajska and Fiejka, 1999; Tomanek, 2005). However, the positioning of the coronary ostia has been shown to vary considerably in wild-type mouse hearts (Fernández et al., 2008) without causing disruption in intra-ventricular CA development, making this possibility less likely.

Finally, these CA stems could, in theory, form by normal means—i.e., peritruncal EC anastomosis—with the failure of the intra-ventricular CAs to mature resulting from an additional, ventricle-specific requirement for CXCL12-CXCR4 signaling. However, when ventricular expression of *Cxcl12* was targeted while expression in the aorta was left mostly intact (*Nkx2.5*-Cre conditional mutants), we did not observe major defects in CA development. This seems to preclude a significant role for ventricular CXCL12 in CA development, although analysis of greater numbers may be required to uncover a more subtle role, e.g., in CA growth.

Targeting of *Cxcl12* expression in the aorta (*Mef2c*-Cre) had a considerably more profound impact on CA development, with absent and mispatterned coronary ostia indicating a disruption of CA stem formation. The reduced penetrance and severity of CA defects seen in the *Mef2c* conditional, compared to full, *Cxcl12* nulls was not unexpected, as patches of low level *Cxcl12* expression remained in and around the OT in addition to the untargeted left ventricular expression. However, it is worth noting that left ventricular *Cxcl12* was not sufficient to rescue LCA formation in a number of conditional mutants, underlining the critical role of OT-specific *Cxcl12* expression in CA stem formation. Intriguingly, one mutant exhibited CAs connected to the pulmonary trunk. This is an unusual phenotype, although a similar scenario has been observed in a rare but serious human congenital cardiac anomaly known as anomalous origin of the LCA from the pulmonary artery (ALPACA) (Hauser, 2005; Wesselhoeft et al., 1968). Peritruncal vessels do not normally penetrate the pulmonary trunk for reasons that are not well understood, but may involve differential gene expression (Bernanke and Velkey, 2002; González-Iriarte et al., 2003). Clearly, expression of *Cxcl12* is insufficient to regulate this differential invasion of the aorta, as it is expressed at equally high levels in the aorta and the pulmonary trunk; therefore, repulsive factors preventing EC migration toward the pulmonary trunk may also contribute. It is not known how such repulsion is overcome in the *Mef2c*-cre *Cxcl12* conditional mutant, but it is possible that uneven recombination of the floxed *Cxcl12* allele in the OT, leading to an altered balance of CXCL12 in the outflow vessels, may play a role. Together with the observation of CA branches from the IVS growing into the ventral portion of the left ventricle (in the absence of a left ostium; Table 1), our results suggest that compensatory mechanisms of CA development are induced when normal CA stem development fails, perhaps facilitated by residual *Cxcl12* expression in the conditional hearts.

SLV defects were always observed in *Cxcl12* knockouts and frequently associated with the conditional null phenotype (*SM22α*-cre, *Mef2c*-Cre, and, to a much lesser extent, *Nkx2.5*-Cre). Although we did not investigate the basis of the valve defects in this study, a role in endothelial-to-mesenchymal transformation (Endo-MT) seems likely, as *Cxcr4* is expressed in the endothelial lining of the cardiac cushions as well as in coronary ECs. The second CXCL12 receptor, CXCR7 (Balabanian et al., 2005; Burns et al., 2006), is also expressed in the endocardial lining of the OT, while SLV defects have been identified in *Cxcr7* mutants (Sierro et al., 2007; Yu et al., 2011). Therefore, a combined role for CXCR4 and CXCR7 in valve formation might be expected.

While this article was in its revision stages, the K. Red-Horse laboratory published a study describing the role of VEGF-C in peritruncal vessel growth and described a capillary plexus they termed the “aortic sub-epicardial vessels,” or ASVs, which circle the lateral side of the aorta, expanding as development proceeds, and connect to the aortic lumen (Chen et al., 2014a). We believe that these ASVs are continuous with the peritruncal vessels we describe, as we have also observed superficial vessels on the ventral side of the aorta connecting to the deeper peritruncal vessels (data not shown). However, we focused our attention more closely on the vessels deep in the aortic wall that, we consider, form the critical connections with the lumen. Chen et al. showed that VEGF-C is required for the initial growth and expansion of the peritruncal vessels and went on to describe a population of CMs specific to the aorta. The CMs are predicted to provide the angiogenic signal required for anastomosis of the peritruncal vessels with the aortic endothelium, as well as playing a role in patterning the CA stems (Chen et al., 2014a). Our results show that while CXCL12 provides an angiogenic signal for peritruncal ECs, its expression is not restricted to this minor population of CMs described; therefore, it is unlikely to be wholly responsible for the fine positioning of the stems. Thus, we reason that the aortic CMs may secrete an additional factor that co-operates with CXCL12 or perhaps serves to antagonize anti-angiogenic signals acting in the walls of the OT, as suggested in Chen et al.’s report. So far, preliminary analyses of *Cxcl12* null OTs have not revealed major differences in the distribution of CMs (data not shown), although further investigation is required. The recent work on VEGF-C adds to previous reports by Tomanek and colleagues of a requirement for VEGF signaling in CA stem formation (Tomanek et al., 2002, 2006). Taken together, the combined data suggest a model of CA stem formation whereby VEGF-C and CXCL12 act sequentially to direct growth and anastomosis of the peritruncal vessels, respectively.

Our results have shown that the CXCL12/CXCR4 signaling pathway plays an essential role in CA development; failure of arteriogenesis in *Cxcl12* and *Cxcr4* mutant hearts most likely explains the lethality caused by loss of either gene. Our data showing the role of CXCL12 in the anastomosis of peritruncal ECs provide insight into the molecular processes that control the earliest stages of CA development, which could potentially inform future therapeutic interventions to regenerate coronary vessels and effect cardiac repair. For instance, sequential, timed activation/repression of multiple signaling pathways, including CXCl12, e.g., using modified RNAs (Zangi et al., 2013), may well be more effective at inducing neovascularization of ischemic tissue than treatment with a single effector.

## Experimental Procedures

### Mutant Mouse Breeding

*Cxcl12* mutants (*Cxcl12-GFP*; Ara et al., 2003) and *Cxcl12*^*fl/fl*^ (Greenbaum et al., 2013), *Cxcr4*^*fl/fl*^ (Nie et al., 2004), *Tie2-Cre* (Kisanuki et al., 2001), *SM22α-Cre* (Holtwick et al., 2002), *Nkx2.5-Cre* (Moses et al., 2001), and *Mef2c-Cre* (Verzi et al., 2005) mice have been described previously. Generation of *Cxcr4* nulls and *Cxcr4* and *Cxcl12* conditional mutants is described in the Supplemental Experimental Procedures. Animal work was carried out according to UK Home Office regulations.

### Immunolabeling

For whole-mount immunolabeling, hearts were permeabilized in PBS with 0.1% Tween 20 (PBST), blocked for 1 hr in PBST/10% goat serum, and incubated overnight at 4°C with primary antibody in blocking buffer. Primary antibodies anti-PECAM-1 (Mec13:3, Pharmingen) and anti-SM22 α (Abcam) were diluted in blocking buffer at 1:50 and 1:250, respectively. Hearts were washed several times in PBST (1-hr washes) before incubation overnight at 4°C with secondary antibody (Alexa Fluor 594 conjugate, Life Technologies). Fluorescent images were captured on a Zeiss Axio Lumar.V12 stereomicroscope.

Immunolabeling of 10-μm paraffin sections and 12-μm frozen sections was carried out according to standard protocols. Briefly, sections were incubated in blocking buffer for 1 hr at room temperature (PBS/10% BSA/1% goat serum for wax sections and PBS/10% BSA/10% goat serum/0.1% Triton X-100 for cryosections) followed by primary antibody in blocking buffer at 4°C overnight. Secondaries were Alexa Fluor conjugated antibodies (Life Technologies, Abcam, Jackson ImmunoResearch), and DNA was counterstained using DAPI (Sigma). Images were captured on a Zeiss Axioimager Z1 microscope. Further details, including details of antigen retrieval and antibodies used, can be found in Supplemental Experimental Procedures.

### HREM

Embryos were bled out for 5 min, and then hearts were dissected out and fixed for 30 min in 4% paraformaldehyde. The hearts were then rocked in a large volume of water for 60 min (with three or four changes of water) to lyse the blood cells and transferred back to fix. HREM was carried out as described elsewhere (Mohun and Weninger, 2012). See also Supplemental Experimental Procedures.

### In Situ Hybridization

In situ hybridization was carried out on 10-μm paraffin sections using standard methods. Hybridization temperature was 70°C. Digoxigenin-labeled probes for *Cxc12* and *Cxcr4* were synthesized from full-length IMAGE clones (IMAGE:3483088 and IMAGE:3592479, respectively). See Supplemental Experimental Procedures for further details.

### Confocal Analysis

Immunolabeled hearts from control (wild-type or *Cxcl12*^*+/−*^) and *Cxcl12*^*−/−*^ (E12.5–E15.5) embryos were dehydrated through a methanol series before clearing with one part benzyl alcohol/two parts benzyl benzoate solution (BABB; Sigma). Embryos were examined by epifluorescence on an inverted LSM710 confocal system mounted on an AxioObserver Z1 microscope (Carl Zeiss, UK). See Supplemental Experimental Procedures for further details.

### Statistical Analysis

When comparing the number of anastomoses formed with the aortic lumen in *Cxcl12* nulls versus controls, we calculated statistical significance using an unpaired Student’s t test. Significance was accepted when p < 0.05.

### Quantification of Relative CA Length

E16.5 hearts in paraffin wax were sectioned at 12 μm and stained with anti-SM22α antibody to label smooth muscle cells. The length of the CAs relative to the total distance from the left or right ostium to the tip of each heart was calculated as follows: the number of sections from the tip of the heart to the left or right coronary ostium was multiplied by the section thickness, giving a “heart length” figure. The number of consecutive sections with visible SM22α-stained arteries in the left or right ventricles was similarly multiplied by the section thickness and subsequently divided by heart length to give a relative measure of CA length.

## Figures and Tables

**Table 1:** CA and SLV Defects in *Mef2c-Cre;Cxcl12*^*fl/−*^ Mutant Hearts

Mutant	LCA	RCA	Ostia	SLV Defects
1	X	✓	right ostium only	✓
2	X	✓	right ostium only	✓
3	X^a^	✓	right ostium only	X
4	X	✓	right ostium only	✓
5	✓	✓	LCA and RCA branch from right ostium	partial^b^
6	✓	✓	LCA and RCA connect to RV and PT, respectively^c^	✓
7–9	✓	✓	left and right ostia	✓

X indicates that no defects were detected. PT, pulmonary trunk; RV, right ventricle.

a CAs were observed in the ventral portion of the LV, branching from a CA located in the IVS.

b Left aortic valve leaflet was normal.

c A third ostium was formed by a CA arising from the IVS and connecting to the aorta.

## Supplemental Information

Document S1. Supplemental Experimental Procedures and Figures S1–S5

Movie S1. HREM Analysis of E17.5 Hearts, Related to Figure 1 Front erosion through E17.5 wild-type (left) and *Cxcl12*^*−/−*^ (right) hearts. Wild-type: note pulmonary valve (PV) visible at 11 s, CAs connecting to aorta at 15 s (OS), and aortic valve (AoV) leaflets from 17 to 19 s. Intra-ventricular arteries are visible from 14 s onward. In the mutant, defective PV and AoV leaflets can be observed from 8 to 12 s and from 17 to 21 s, respectively. Note that the aorta overrides a VSD (19 s). A single, short CA can be observed on the ventral side of the aorta (14–15 s), and the ostium it connects to (OS) is both laterally and longitudinally mis-positioned (15 s). Intra-ventricular arteries are absent.

Document S2. Article plus Supplemental Information
