# Supplementary material for: The CXCL12/CXCR4 Axis Plays a Critical Role in Coronary Artery Development
Source: Dev Cell. 2015 May 26;33(4):455–68. doi: 10.1016/j.devcel.2015.03.026 (PMC4448146; doi:10.1016/j.devcel.2015.03.026)
Supplement: Document S1. Supplemental Experimental Procedures and Figures S1–S5 [file mmc1.pdf]

Developmental Cell
Supplemental Information

# The CXCL12/CXCR4 Axis Plays a Critical Role in Coronary Artery Development

Sarah Ivins, Joel Chappell, Bertrand Vernay, Jenifer Suntharalingham, Alexandrine Martineau, Timothy J. Mohun, and Peter J. Scambler

# Inventory of Supplemental Information

Supplemental Figures S1-S5

**Figure S1, related to Figure 1.** Coronary artery and ostia positioning defects in *Cxcl12* mutants.

**Figure S2, related to Figure2.** Deletion of *Cxcr4* in the endothelial lineage results in coronary ostia and SLV defects.

**Figure S3, related to Figure 3.** Deletion of *Cxcr4* in the vSMC lineage results in SLV defects but smooth muscle coverage of CAs is unaffected.

**Figure S4, related to Figure 5.** Confocal analysis of the peritruncal plexus in *Cxcl12* mutants at E13.5-E14.5.

**Figure S5, related to Figure 6.** Variable SLV and CA defects in *Mef2c-Cre;Cxcl12*$^{fl/-}$ hearts.

**Legend for Movie S1, related to Figure1**. HREM analysis of E17.5 hearts.
Supplemental Experimental procedures
References

## Figure S1

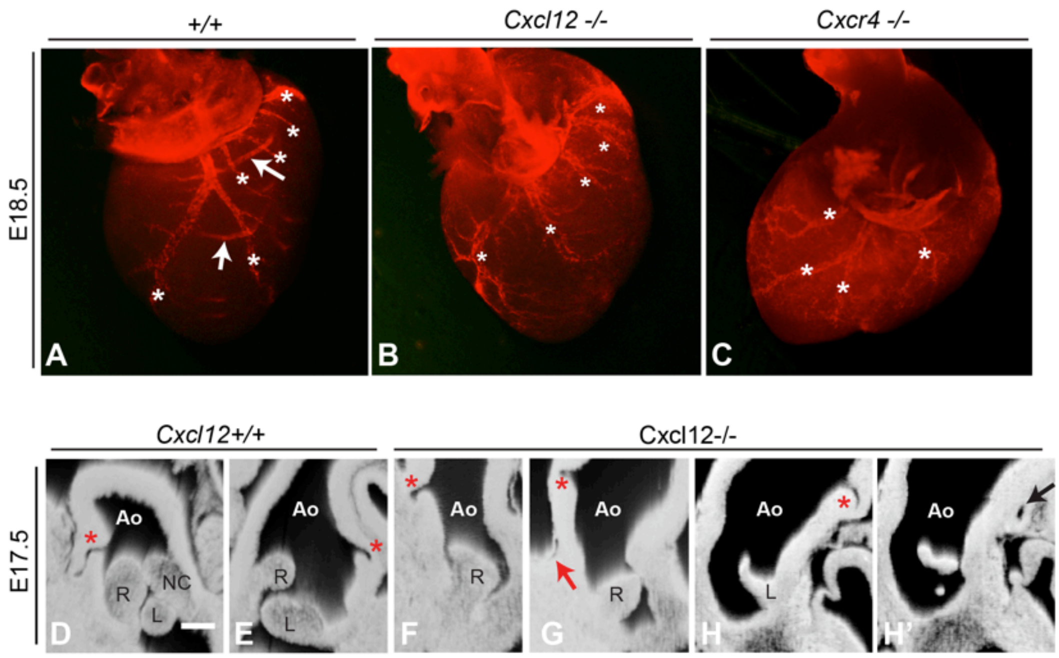


**Figure S1, related to Figure 1. Coronary artery and ostia positioning defects in *Cxcl12* mutants**

(A-C) Wholemount labelling of E18.5 hearts with anti-SM22α antibody. The right CA (arrows in A) is absent in both *Cxcl12* and *Cxcr4* null hearts. Asterisks indicate coronary veins on the dorsal surface of the heart. (D-H') HREM analysis of ostia defects in *Cxcl12*$^{-/-}$ hearts (dynamic reslice of aortic region in ImageJ). Asterisks indicate coronary ostia in wild type control (right and left ostia in D and E respectively). Three nulls (F-H) each have a single, distally positioned ostium (asterisks). The red arrow in heart (G) shows the sub-epicardial location of its proximal CA. In (H) and (H') the distal ostium gives rise to a short stretch of CA that passes along the exterior surface of the aorta (black arrow in H').

Scale bars, 100μm. Ao, aorta; CA, coronary artery; coronary leaflets: L (left), R (right) and NC (non-coronary).

## Figure S2

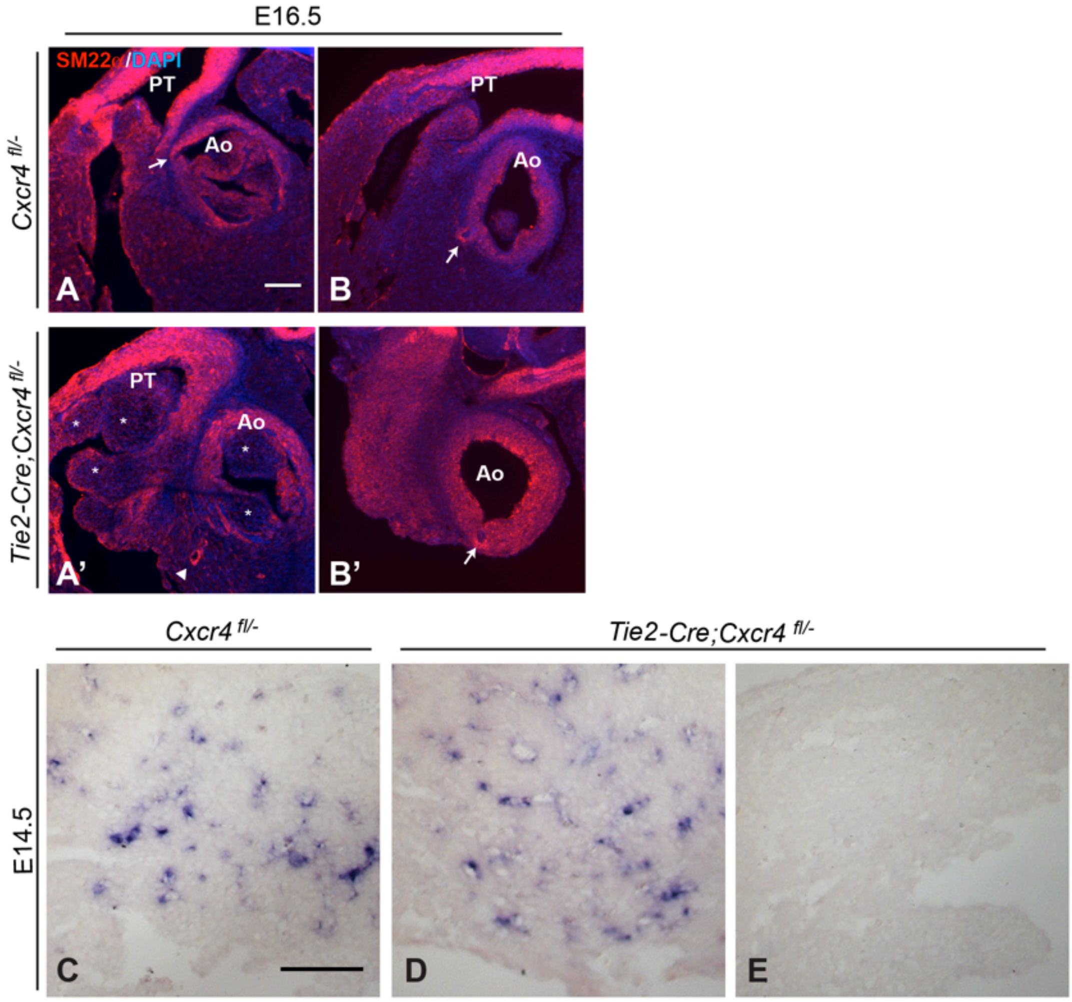


**Figure S2, related to Figure2. Deletion of *Cxcr4* in the endothelial lineage results in coronary ostia and SLV defects**

(A-B') SM22$\alpha$-labelling of OT regions of control and *Cxcr4* conditional null E16.5 hearts. Arrows in (A) and (B) indicate coronary ostia in control heart; the ostium shown in (B) is slightly distal to the aortic valve. Defective SLVs (asterisks indicate valve leaflets) are clearly visible in the *Tie2-Cre;Cxcr4*$^{fl/-}$ heart (A'); arrowhead in (A') indicates a single coronary artery (ostium shown in B'). (C-E) *Cxcr4* expression in the ventricular septal region of E14.5 control and conditional null hearts (*in situ* hybridisation). Note *Cxcr4* expression was variably down-regulated as high levels of *Cxcr4* were present in the *Tie2-Cre;Cxcr4*$^{fl/-}$ heart shown in (D), while in other cases there was virtually no detectable expression (E). This likely accounts for the variability of phenotype observed in this series of conditional mutants. Scale bars, 100$\mu$m. Ao, aorta, OT, outflow tract; PT, pulmonary trunk; SLV, semi-lunar valve.

## Figure S3

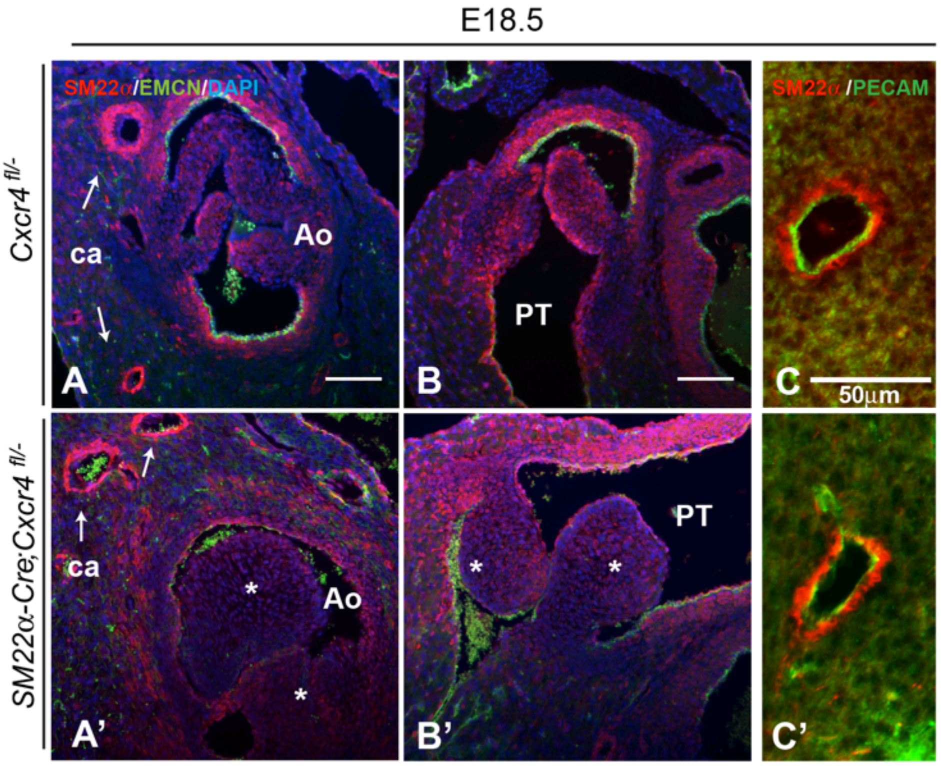


**Figure S3, related to Figure 3. Deletion of *Cxcr4* in the vSMC lineage results in SLV defects but smooth muscle coverage of CAs is unaffected.**

(A-B') SM22α/EMCN-labelling of OT regions of control (*Cxcr4*$^{fl/-}$) and *SM22αCre;Cxcr4*$^{fl/-}$ E16.5 hearts (transverse sections). Asterisks in (A') and (B') indicate defective valve leaflets in the aorta (Ao) and pulmonary trunk (PT) of the *SM22αCre;Cxcr4*$^{fl/-}$ heart respectively. Arrows indicate CAs. (C, C') PECAM/SM22 staining shows CA coverage with smooth muscle cells is unimpaired in *SM22αCre;Cxcr4*$^{fl/-}$ hearts.

Scale bars, 100µm in (A) and (B), otherwise as indicated. CAs, coronary arteries; ECMN, endomucin; OT, outflow tract.

## Figure S4

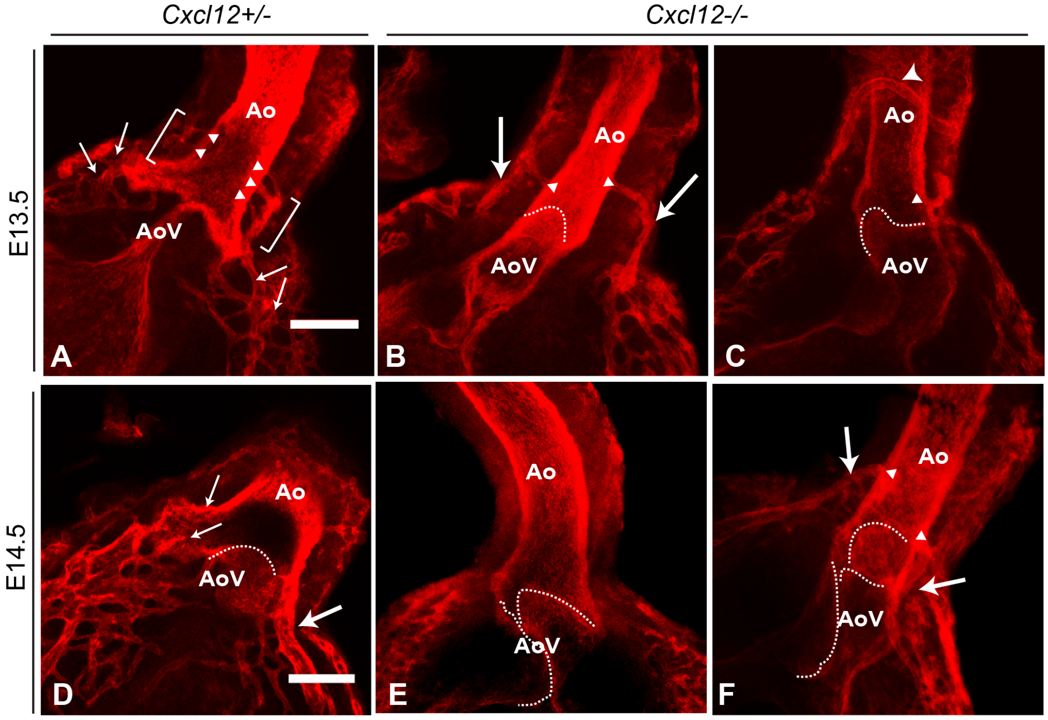


**Figure S4, related to Figure 5. Confocal analysis of the peritruncal plexus in *Cxcl12* mutants at E13.5-E14.5.**

(A-F) Confocal microscopic images of E13.5-E14.5 wholemount PECAM-labelled control and *Cxcl12*$^{-/-}$ hearts showing the aorta (Ao) and peritruncal plexus (maximum intensity z-projections of confocal stacks). For clarity, dotted lines have been used to delineate the edges of the aortic valves (AoV). Arrowheads indicate points of contact between ECs/vessels and aortic lumen. (A-C) At E13.5 the control peritruncal plexus (bracketed region in A) still forms multiple contacts with the aortic lumen (arrowheads), and is connected to the intra-ventricular coronary plexus (small arrows). (B) shows a Cxcl12$^{-/-}$ aorta with sub-epicardially-localised vessels (arrows) forming single, distal connections on either side of the aortic lumen (arrowheads). In (C) an ectopic vessel region crosses over the aortic lumen (large arrowhead) and a wide, lumenised vessel buds from one side of the aorta (small arrowhead). By E14.5 the peritruncal plexus is disappearing (D). The large arrow in (D) shows a single major vessel connecting on left side of the control aorta; two smaller vessels are connected to the right side (small arrows). The *Cxcl12*$^{-/-}$ in (E) lacks any vessels in the aorta, whilst in (F), vessels connect either side of the aorta (arrows) but fail to branch into the ventricles. Scale bars, 100μm.

## Figure S5

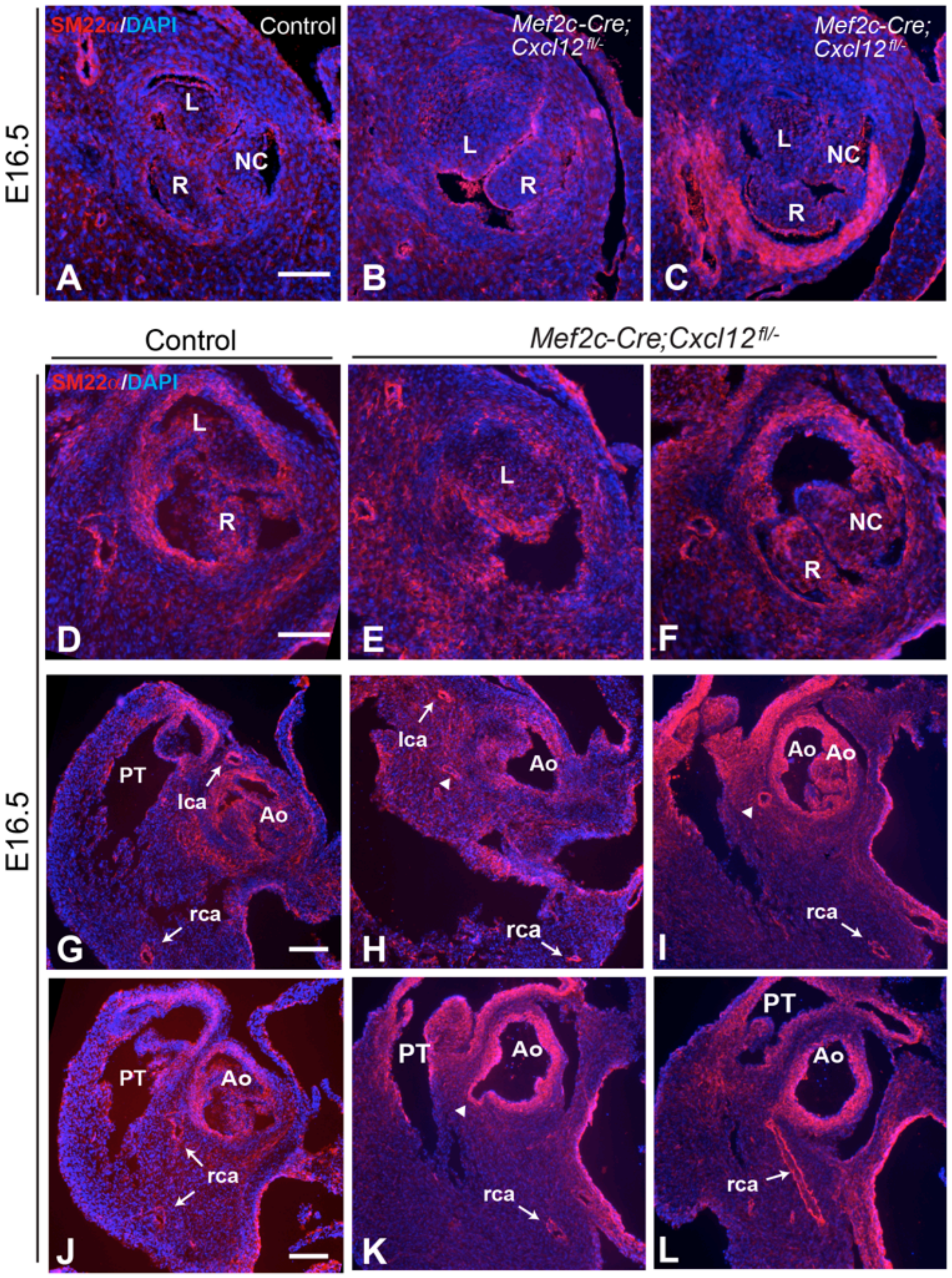


**Figure S5, related to Figure 6. Variable SLV and CA defects in *Mef2c-Cre;Cxcl12$^{fl/-}$* hearts.**

(A-C) Anti-SM22α antibody labelling of E16.5 sections through the aorta; CA defects are not always accompanied by aortic valve defects in *Mef2c-Cre;Cxcl12$^{fl/-}$* hearts. The conditional null in (B) shows thickened left (L) and right (R) aortic valve leaflets whereas in another null (C) the valve leaflets

appear normal. (D-F) A further null shows a combination of thickened (E) and normal (F) valve leaflets. In the same conditional null both left and right CAs are present (lca, rca, arrows in H, I, K, L) as well as a CA branching into the IVS (arrowhead in H). However only a single ostium (arrowhead) is formed on the right side (K). Corresponding control sections are shown in (G) and (J).

Scale bars, 100μm. Ao, aorta; coronary valve leaflets: left (L), right (R) and non-coronary (NC); IVS, inter-ventricular septum; PT, pulmonary trunk.

**Movie S1, Related to Figure1. HREM analysis of E17.5 hearts.**

Front erosion through E17.5 wild type (left) and *Cxcl12*$^{-/-}$ (right) hearts. Wild type: note pulmonary valve (PV) visible at 11s, coronary arteries connecting to aorta at 15s (OS), and aortic valve (AoV) leaflets from 17-19s. Intra-ventricular arteries are visible from 14s onwards. In the mutant, defective pulmonary and aortic valve leaflets (PV and AoV) can be observed from 8-12s and 17-21s respectively. Note that the aorta over-rides a VSD (19s). A single, short coronary artery can be observed on the ventral side of the aorta (14-15s), the ostium it connects to (OS) is both laterally and longitudinally mis-positioned (15s). Intra-ventricular arteries are absent.

## Supplemental Experimental Procedures

### Mutant mouse breeding

*Cxcr4* null mice were obtained by crossing *Cxcr4* $^{fl/fl}$ mice with βactin-Cre mice (Lewandoski et al., 1997). Offspring carrying both *Cre* and recombined *Cxcr4* alleles were bred again with wild types to obtain offspring carrying only the recombined *Cxcr4* allele. Mice carrying two copies of the recombined *Cxcr4* allele are referred to in the text as *Cxcr4*$^{-/-}$ or *Cxcr4* null. *Tie2-Cre*;*Cxcr4* $^{fl/-}$mice were generated by crossing *Tie2-Cre;Cxcr4*$^{+/-}$ mice with *Cxcr4* $^{fl/fl}$ mice and *SM22a-Cre*; *Cxcr4*$^{fl/-}$ mice by crossing *SM22a-Cre*; *Cxcr4*$^{+/-}$ mice with *Cxcr4* $^{fl/fl}$. Similarly, *Nkx2.5-Cre;Cxcl12*$^{fl/-}$ and *Mef2c-Cre;Cxcl12*$^{fl/-}$ conditionals were generated by crossing *Cxcl12* $^{fl/fl}$ mice with *Nkx2.5-Cre* ;*Cxcl12*$^{+/-}$ and *Mef2c-Cre* ;*Cxcl12*$^{+/-}$ mice respectively. All mice were maintained on a C57Bl/6 background. Animal work was carried out according to UK Home Office regulations.

### Fixation of embryonic hearts

Whole-mount hearts were fixed in 4% paraformaldehyde (PFA) for 15-30 minutes. For immunolabelling or *in situ* hybridisation on paraffin sections, hearts were fixed overnight in 10% neutral-buffered formalin or 4% PFA respectively at 4°C. For frozen sections, hearts were fixed for 15-60 minutes in 4% PFA, incubated overnight in 30% sucrose at 4°C, then mounted in optical cutting temperature compound (OCT, RA Lamb) and snap-frozen.

### Immunolabelling

For paraffin sections, heat-induced antigen retrieval in Target Retrieval Solution Citrate pH 6 (Dako) was carried out after de-paraffinisation and rehydration, and

sections were blocked for one hour. The following antibodies were used: anti-PECAM (Abcam) at 1:50, anti-endomucin (EMCN) (V.737, Santa Cruz), anti-SM22$\alpha$ as above, and anti-NOTCH1 (Cell Signalling) at 1:400. Signal was detected using Alexa Fluor®-594 and 488 conjugated secondary antibodies (Life Technologies) at 1:500. For PECAM staining in combination with anti-SM22, rat anti-CD31 (RM0032-1D12, Abcam) was used at 1:100. In this case, antigen retrieval was carried out by proteinase K digestion for 30 minutes at room temperature (10$\mu$g/ml). The PECAM signal was amplified using anti-rat biotin-conjugated secondary (Thermo Scientific Pierce) at 1:500 followed by Streptavidin, Alexa Fluor® 594 conjugate (Life Technologies).

Frozen sections were permeabilised for 5 minutes in 0.5% Triton X-100 (Sigma) prior to blocking in 10% BSA/10% goat serum/0.1% Triton X-100 in PBS). Anti-CXCR4 (UMB2, Abcam) was used at 1:300 and anti-PECAM (MA3105, Thermo Scientific Pierce) at 1:400. The following anti-Armenian Hamster secondary antibodies were used to detect PECAM signal: Alexa Fluor 594 conjugate (Jackson ImmunoResearch) at 1:400 and Alexa Fluor 488 conjugate (Abcam) at 1:500. All incubation of sections with primary antibodies were carried out at 4°C overnight. Staining with secondaries was for one hour at room temperature; washes were carried out using PBS.

***In situ* hybridisation**

Paraffin sections were de-waxed (Histoclear, National Diagnostics) and re-hydrated through an ethanol series. Sections were permeablilised using Proteinase K (Sigma) at 20$\mu$g/ml for 8 min. This was followed by glycine at 2mg/ml for 5 min, washing in PBS and post-fixing in 4% paraformaldehyde for 20 min. After rinsing in PBS,

sections were pre-hybridised in hybridisation buffer (50% de-ionised formamide (Promega), 5xSSC pH 5.0, 50μg/ml yeast tRNA (Sigma), 1% SDS and 50 μg/ml heparin (Sigma)) for one hour at 70°C. Probe incubation was carried out at 70°C overnight followed by washes at 65°C as follows: 3x 15 min in 50% formamide/5x SSC/1% SDS and 2 x 15 min in 50% formamide/2x SSC. The samples were allowed to cool to room temperature then washed twice for 10min each in MABT (100mM maleic acid, 150mM NaCl, 0.1% Tween-20, pH 7.5) with 2mM tetramisole hydrochloride. (levamisol, Sigma). Blocking was carried out in 2% Blocking Reagent (Roche Life Science)/5% sheep serum/5% goat serum (in MABT) for one hour at room temperature before incubating with anti-Digoxygenin-AP antibody at 1:2000 (4°C overnight). After removal of the antibody the sections were washed in MABT/levamisol and equilibrated in alkaline phosphatase buffer (2 x 5 min washes) before staining with BM Purple solution (Roche Life Science)>

**High Resolution Episcopic Microscopy (HREM)**

For further details of HREM see (Mohun and Weninger, 2012a) and (Mohun and Weninger, 2012b). Movies were created using Osirix software.

**3D reconstruction**

Image stacks containing the aorta were cropped in Fiji (Schindelin et al., 2012) and the 3D reconstructions were created using Imaris (Bitplane AG) surpass module with the projection mode set to blend. Snapshots of similar 3D orientation were selected for the figures.

**Confocal Analysis**

For whole-mount hearts, images were captured with a LD LCI Plan-Apochromat 25x/NA 0.8 water immersion DIC objective or a LD LCI Plan-Apochromat 10x/NA 0.3 air DIC objective (Carl Zeiss Ltd, United Kingdom). The Alexa Fluor 594 dye was excited with a 594 nm diode. The pinhole aperture was set to image a 3.4 µm thick optical section and z-stack images were acquired with 1.7-2.2 µm spacing between optical slices. The images files were exported into ImageJ (Rasband, 1997-2009, http://rsb.info.nih.gov/ij/) where they were processed for publication.

For analysis of sections, Alexa Fluor® 488 and Alexa Fluor® 594 dyes were sequentially excited with a 488 nm Argon laser and 561nm diode, and images were captured with the LD LCI Plan-Apochromat 10x/NA 0.3 air DIC objective. For DAPI and Alexa Fluor® 594-stained sections, 405 nm and 561nm laser diodes were used; images were captured with the Zeiss Plan-Apochromat 20x/0.8 M27 air objective.

## Supplemental References

Lewandoski, M., Meyers, E.N., and Martin, G.R. (1997). Analysis of Fgf8 gene function in vertebrate development. Cold Spring Harb Symp Quant Biol *62*, 159-168.

Mohun, T.J., and Weninger, W.J. (2012a). Episcopic three-dimensional imaging of embryos. Cold Spring Harb Protoc *2012*, 641-646.

Mohun, T.J., and Weninger, W.J. (2012b). Generation of volume data by episcopic three-dimensional imaging of embryos. Cold Spring Harb Protoc *2012*, 681-682.

Schindelin, J., Arganda-Carreras, I., Frise, E., Kaynig, V., Longair, M., Pietzsch, T., Preibisch, S., Rueden, C., Saalfeld, S., Schmid, B., *et al.* (2012). Fiji: an open-source platform for biological-image analysis. Nat Methods *9*, 676-682.
