# Supplementary figures and images for: The CXCL12/CXCR4 Axis Plays a Critical Role in Coronary Artery Development
Source: Dev Cell. 2015 May 26;33(4):455–68. doi: 10.1016/j.devcel.2015.03.026 (PMC4448146; doi:10.1016/j.devcel.2015.03.026)

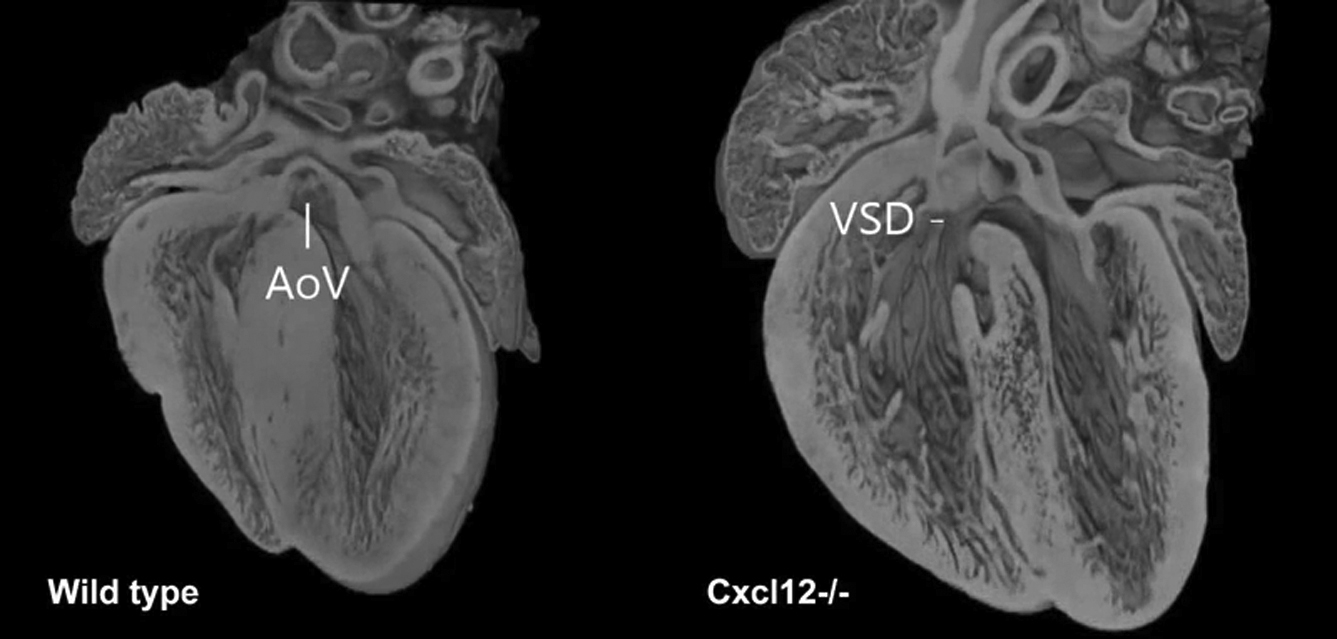

Supplement: Movie S1. HREM Analysis of E17.5 Hearts, Related to Figure 1 — Front erosion through E17.5 wild-type (left) and Cxcl12−/− (right) hearts. Wild-type: note pulmonary valve (PV) visible at 11 s, CAs connecting to aorta at 15 s (OS), and aortic valve (AoV) leaflets from 17 to 19 s. Intra-ventricular arteries are visible from 14 s onward. In the mutant, defective PV and AoV leaflets can be observed from 8 to 12 s and from 17 to 21 s, respectively. Note that the aorta overrides a VSD (19 s). A single, short CA can be observed on the ventral side of the aorta (14–15 s), and the ostium it connects to (OS) is both laterally and longitudinally mis-positioned (15 s). Intra-ventricular arteries are absent. [file mmc2.jpg]
